# Giant pedunculated lipoma of the colon causing colo-colic intussusception in a young adult woman: a case report and review of the literature

**DOI:** 10.1093/jscr/rjag794

**Published:** 2026-09-03

**Authors:** Aziz Atallah, Zied Hadrich, Mohamed Hajri, Montassar Zaafouri, Rached Bayar, Sahir Omrani

**Affiliations:** Department of Visceral Surgery, Mongi Slim Hospital, Marsa, Tunisia; University of Tunis El Manar, Faculty of Medicine of Tunis, Tunis, Tunisia; Department of Visceral Surgery, Mongi Slim Hospital, Marsa, Tunisia; University of Tunis El Manar, Faculty of Medicine of Tunis, Tunis, Tunisia; Department of Visceral Surgery, Mongi Slim Hospital, Marsa, Tunisia; University of Tunis El Manar, Faculty of Medicine of Tunis, Tunis, Tunisia; Department of Visceral Surgery, Mongi Slim Hospital, Marsa, Tunisia; University of Tunis El Manar, Faculty of Medicine of Tunis, Tunis, Tunisia; Department of Visceral Surgery, Mongi Slim Hospital, Marsa, Tunisia; University of Tunis El Manar, Faculty of Medicine of Tunis, Tunis, Tunisia; Department of Visceral Surgery, Mongi Slim Hospital, Marsa, Tunisia; University of Tunis El Manar, Faculty of Medicine of Tunis, Tunis, Tunisia

**Keywords:** colonic lipoma, intussusception, intestinal obstruction, right hemicolectomy, adult, case report

## Abstract

Colonic lipomas are uncommon benign submucosal tumours that are usually asymptomatic, but large lesions may act as a lead point for intussusception. We report a 36-year-old woman admitted with 2 days of epigastric pain and vomiting. Inflammatory markers were mildly raised and contrast-enhanced computed tomography showed a colo-colic intussusception of the transverse colon with an endoluminal fat-density mass as the lead point, without proximal distension. Because of the risk of bowel ischaemia she underwent urgent laparotomy; the intussusception reduced spontaneously and a right hemicolectomy with ileocolic anastomosis was performed. Recovery was uneventful and she was discharged on the fourth postoperative day. Histopathology confirmed a benign pedunculated lipoma (5.5 cm) with surface ulceration and no malignancy. Set against the published literature, this case shows that colonic lipoma should be considered in adult intussusception and that resection is both diagnostic and curative.

## Introduction

Lipomas are among the most common benign non-epithelial tumours of the colon, yet they remain clinically silent in the great majority of patients; their reported frequency ranges from 0.2% to 4.4% of benign colonic tumours, with a predilection for the right colon [1, 2]. Lesions larger than ~4 cm are far more likely to become symptomatic, producing abdominal pain, altered bowel habit, bleeding or, less often, mechanical complications [1]. Intussusception is an uncommon cause of intestinal obstruction in adults, accounting for roughly 1% of cases, and—unlike in children—a pathological lead point is identified in most patients, a substantial proportion of which are malignant [3]. Colonic lipoma is one of the commonest benign lead points, but its preoperative distinction from a neoplasm is difficult, so surgical resection is usually required for both treatment and diagnosis [4, 5]. We report a young woman with a giant pedunculated colonic lipoma that caused a colo-colic intussusception, managed by right hemicolectomy, and we review comparable cases from the recent literature.

## Case report

A 36-year-old woman was admitted with epigastric abdominal pain of 2 days’ duration associated with vomiting. There was no fever and no change in bowel habit. Her only relevant history was an open appendicectomy through a McBurney incision 5 years earlier. On examination she was afebrile and in good general condition; the abdomen was soft and depressible with epigastric tenderness and no palpable mass. Laboratory tests showed a C-reactive protein of 51 mg/L and a white-cell count of 8600/mm^3^, with a normal serum lipase (10 IU/L) and normal liver function tests.

**Figure 1:**
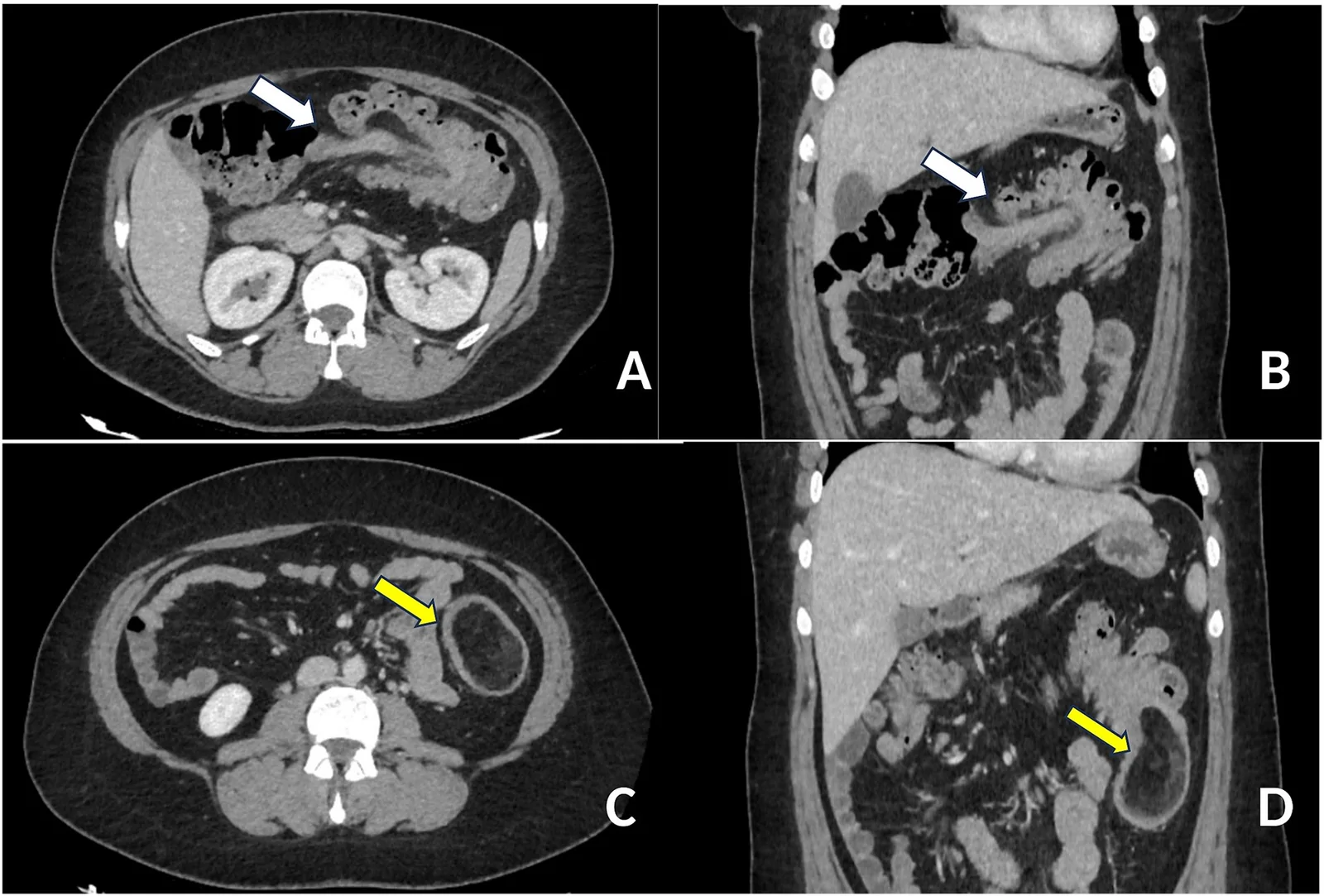
Preoperative contrast-enhanced abdominal CT. Axial (A) and coronal (B) images show the colo-colic intussusception of the transverse colon (white arrow); axial (C) and coronal (D) images show the endoluminal fat-density mass acting as the lead point (yellow arrow).

Contrast-enhanced abdominal computed tomography (CT) demonstrated a colo-colic intussusception of the transverse colon with an endoluminal fat-density mass measuring 64 × 38 × 38 mm acting as the lead point, without an enhancement defect of the bowel wall and without proximal colonic distension (Fig. 1). The intussusception drew the right colon medially, with ascent of the caecum to the right hypochondrium. No other lesion was identified.

**Figure 2:**
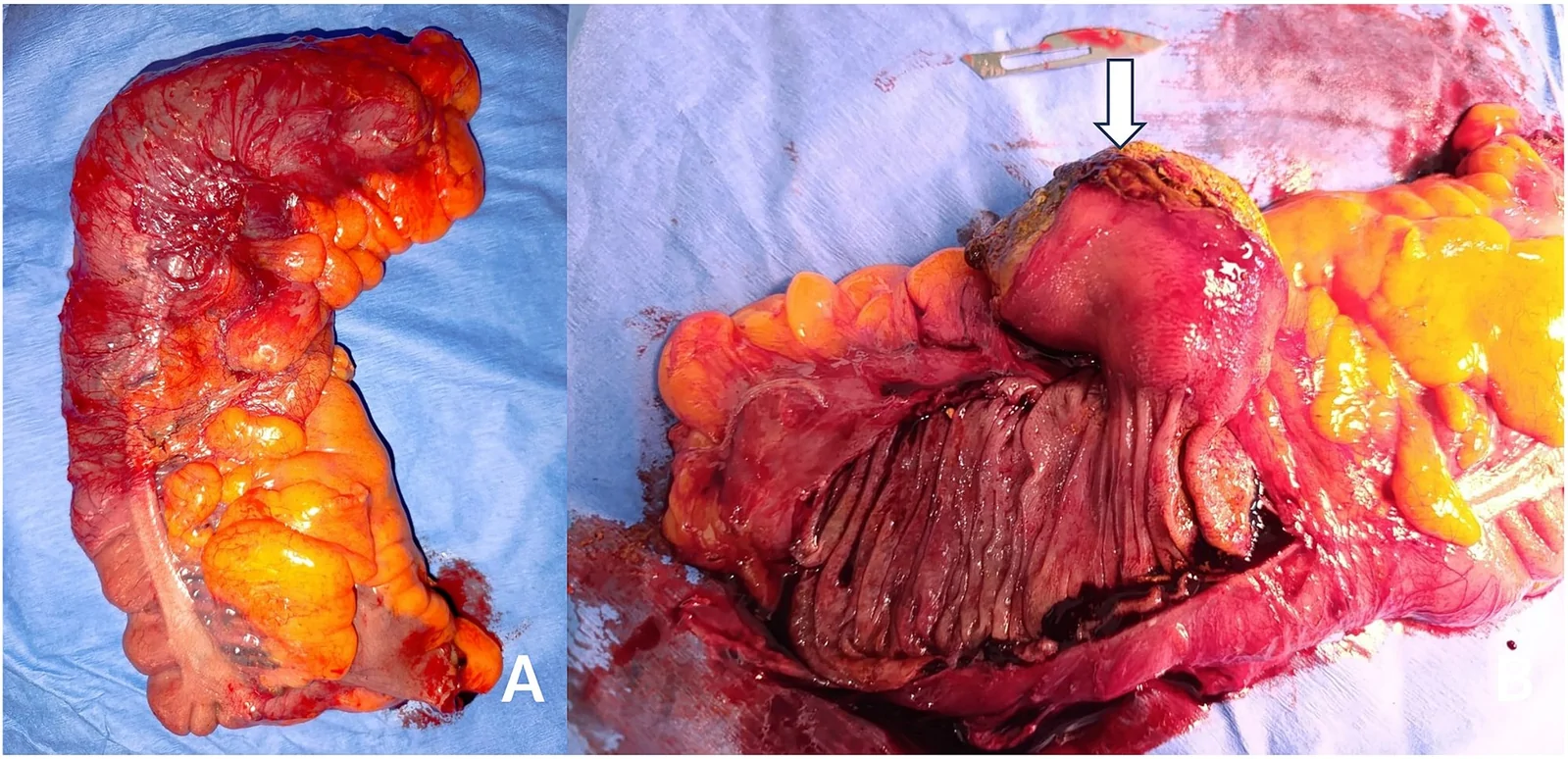
Intraoperative images. (A) External view of the resected right hemicolectomy specimen; (B) opened specimen showing the soft, pedunculated lipomatous intraluminal mass (arrow) with an extensively ulcerated surface.

In view of the risk of colonic ischaemia, the patient was operated on urgently after brief resuscitation. Through a midline laparotomy, a colo-colic intussusception was found in the transverse colon, 15 cm from the left colic angle; the remaining colon was non-distended and well vascularized, and the caecum lay in a subhepatic position. The intussusception reduced spontaneously during manipulation of the colon, and the mass was palpated ~15 cm from the caecum. A right hemicolectomy was performed with a hand-sewn termino-lateral ileocolic anastomosis using 4/0 polydioxanone. Estimated blood loss was 50 ml. Opening of the specimen revealed a soft, pedunculated intraluminal formation measuring 6 cm (Fig. 2A and B).

The postoperative course was uneventful; oral intake was resumed on the first postoperative day and the patient was discharged on the fourth postoperative day. Histopathological examination of the right hemicolectomy specimen (3 cm of ileum and 17 cm of colon) showed a pedunculated polypoid mass of 5.5 × 5.5 × 4 cm, yellowish and lipomatous on sectioning, lying 8 cm from the ileocaecal valve. The lesion was composed of lobules of mature adipocytes without cytological atypia, separated by fibrous septa, with a richly vascularized subserosal pedicle; the overlying mucosa was extensively ulcerated, atrophic and focally necrotic. The resection margins were free, the sampled lymph nodes were reactive, and there were no histological features of malignancy. At the last follow-up, 2 years after surgery, the patient remained asymptomatic with no recurrence.

## Discussion

Colonic lipomas are slow-growing submucosal tumours of mature adipose tissue that usually remain asymptomatic; symptoms correlate chiefly with size, lesions >4 cm being the most likely to bleed, obstruct or, as here, initiate an intussusception [1, 2]. In adults an organic lead point underlies most intussusceptions, and because malignancy cannot be excluded preoperatively, resection is the accepted standard [3, 4]. Our patient illustrates the classic mechanism: a large pedunculated lipoma telescoping the bowel, with secondary mucosal ulceration from traction and ischaemia, exactly as seen on the specimen.

Although colonic lipomas are typically reported in the fifth to seventh decades, comparable cases in younger adults are well documented, including women in their thirties and forties (Table 1), so the present case is not exceptional for age [6, 7]. CT is central to diagnosis: a well-circumscribed endoluminal mass of fat density (negative Hounsfield units) is highly suggestive, yet overlap with other submucosal lesions and with malignancy persists, and lipomas have been mistaken for stromal tumours [5]. This diagnostic uncertainty explains why most authors, and our team, proceed to a formal oncological resection rather than local excision when the diagnosis is not histologically secured [4, 8].

**Table 1:** Present case and representative published cases of colonic lipoma causing intussusception or obstruction in adults

Reference (year)	Age/Sex	Site	Size (cm)	Mechanism / presentation	Procedure	Outcome
Casiraghi *et al*., 2016 [10]	47/F	Caecum	NR	Ileocolic + colocolic intussusception, obstruction	Surgical resection	Uneventful
Pichioni *et al*., 2023 [1]	65/F	Descending colon	2.6	Luminal obstruction (no intussusception)	Laparoscopic segmental colectomy	Uneventful
Kyaw *et al*., 2023 [11]	NR	Colon	Large	Intussusception (lipoma lead point)	Surgical resection	Uneventful
Angelakakis *et al*., 2024 [6]	30/F	Caecum/transverse	6.7	Ileocolic intussusception	Right hemicolectomy	Uneventful
Suleiman *et al*., 2024 [12]	F (NR)	Transverse colon	6 × 5	Colo-colic intussusception	Resection + double-barrel colostomy	Uneventful
Zubi & Atiyah, 2025 [7]	42/F	Colon	5	Colo-colic intussusception	Open segmental colectomy	Discharged POD 8
Alsuhaimi *et al*., 2025 [13]	52/M	Transverse colon	NR	Cecocolic intussusception	Limited right hemicolectomy	Symptom-free at 3 months
Jha *et al*., 2025 [8]	65/F	Ascending colon	NR	Colo-colic intussusception	Laparoscopic right hemicolectomy	Uneventful
Present case	36/F	Right / proximal transverse colon	5.5	Colo-colic intussusception	Right hemicolectomy	Discharged POD 4

F, female; M, male; NR, not reported; POD, postoperative day. Numbers in parentheses correspond to the reference list of the manuscript.

The role of reducing the intussusception before resection remains debated, since reduction may risk perforation or seeding if the lead point is malignant; many therefore advocate resection without prior reduction, although spontaneous reduction—as occurred here—does not alter the indication for resection [3, 9]. Across comparable adult cases summarized in Table 1, right or segmental colectomy consistently achieves both cure and definitive diagnosis with low morbidity [6–8, 10–13]. The main limitation of this report is inherent to its single-case design; reassuringly, the patient remained asymptomatic and recurrence-free at 2 years of follow-up.

In conclusion, colonic lipoma should be considered in any adult presenting with intussusception or unexplained large-bowel obstruction. Cross-sectional imaging guides the diagnosis, and surgical resection is both therapeutic and diagnostic.

## Data Availability

All data supporting the findings of this report are included within the article.
